# Supplementary material for: Global Proteomics Investigation of SAMT-247 Targets: An Antiviral Thioester that Acetylates Zinc Finger Proteins
Source: bioRxiv. 2026 Apr 30:2026.04.28.721345. Preprint. [Version 1] doi: 10.64898/2026.04.28.721345 (PMC13142373; doi:10.64898/2026.04.28.721345)

Supplemental Figure 4. Modification of His-MGMT in the absence or presence of SAMT-247. The green line indicates the sequence coverage of His-MGMT in the DMSO-treated control and the red line in the SAMT-247 reactions. Green “A” indicates identification a site of acetylation in the DMSO control and red “A” a site in the SAMT-247 reaction. Bold font represents sites with >5-fold increase in acetylation in the SAMT-247 reaction as compared to the DMSO control.

Supplemental Figure 4

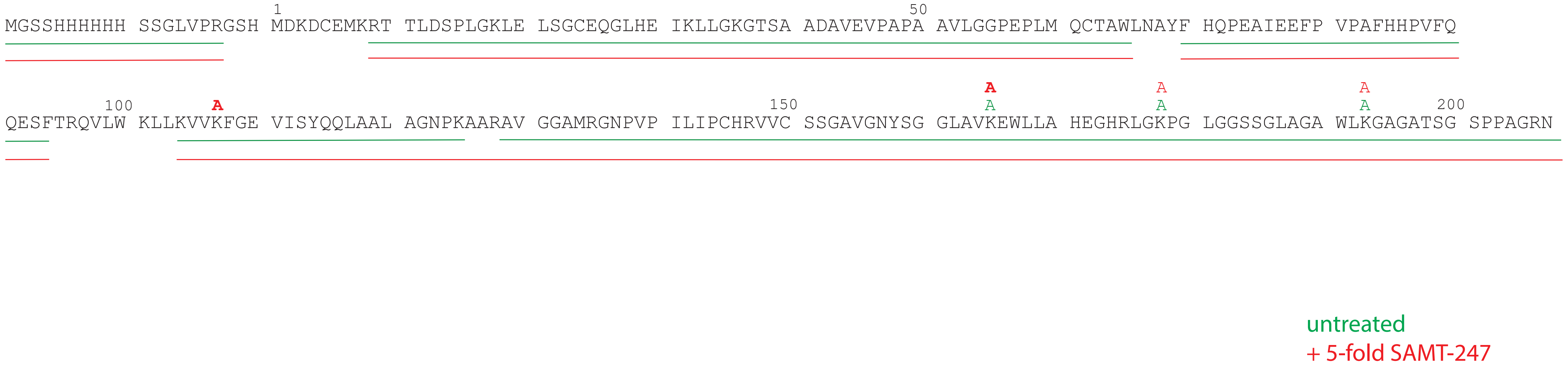

Supplement: Supplement 1 [file media-1.pdf]
