# Supplementary material for: Global Proteomics Investigation of SAMT-247 Targets: An Antiviral Thioester that Acetylates Zinc Finger Proteins
Source: bioRxiv. 2026 Apr 30:2026.04.28.721345. Preprint. [Version 1] doi: 10.64898/2026.04.28.721345 (PMC13142373; doi:10.64898/2026.04.28.721345)
Supplement: Supplement 2 [file media-2.pdf]

Supplemental Figure 3. Modification of ZC3H7A$_{440\text{-}971}$ incubated in THP-1 lysate in the absence or presence of SAMT-247. The red line indicates the sequence coverage of ZC3H7A$_{440\text{-}971}$ in the SAMT-247 reaction. Red "A" indicates sites of acetylation, consistent with SAMT-247 reaction. Yellow highlighting indicates the four zinc finger domains.

Supplemental Figure 3

```
                 A                 A
HELRQACQIC FVKSGPKLMD FTYHANIDHK CKKDILIGRI KNVEDKSWKK

                  A
IRPRPTKTNY EGPYYICKDV AAEEECRYSG HCTFAYCQEE IDVWTLERKG

             A                       A    A
AFSREAFFGG NGKINLTVFK LLQEHLGEFI FLCEKCFDHK PRMISKRNKD

NSTACSHPVT KHEFEDNKCL VHILRETTVK YSKIRSFHGQ CQLDLCRHEV

   A                                      A
RYGCLREDEC FYAHSLVELK VWIMQNETGI SHDAIAQESK RYWQNLEANV

                  A                    A
PGAQVLGNQI MPGFLNMKIK FVCAQCLRNG QVIEPDKNRK YCSAKARHSW

                A           A                 A
TKDRRAMRVM SIERKKWMNI RPLPTKKQMP LQFDLCNHIA SGKKCQYVGN

CSFAHSPEER EVWTYMKENG IQDMEQFYEL WLKSQKNEKS EDIASQSNKE

                                 A              A
NGKQIHMPTD YAEVTVDFHC WMCGKNCNSE KQWQGHISSE KHKEKVFHTE

DDQYCWQHRF PTGYFSICDR YMNGTCPEGN SCKFAHGNAE LHEWEERRDA

         A              A
LKMKLNKARK DHLIGPNDND FGKYSFLFKD LN
```
