# Supplementary material for: Global Proteomics Investigation of SAMT-247 Targets: An Antiviral Thioester that Acetylates Zinc Finger Proteins
Source: bioRxiv. 2026 Apr 30:2026.04.28.721345. Preprint. [Version 1] doi: 10.64898/2026.04.28.721345 (PMC13142373; doi:10.64898/2026.04.28.721345)
Supplement: Supplement 3 [file media-3.pdf]

Supplemental Figure 2. Modification of ZC3H7A$_{440\text{-}971}$ in the absence or presence of SAMT-247. The green line indicates the sequence coverage of ZC3H7A$_{440\text{-}971}$ in the DMSO-treated control and the red line in the SAMT-247 reactions. Green “A” indicates identification a site of acetylation in the DMSO control and red “A” a site in the SAMT-247 reaction. Bold font represents sites with >5-fold increase in acetylation in the SAMT-247 reaction as compared to the DMSO control. Yellow highlighting indicates the four zinc finger domains.

Supplemental Figure 2

```
HELRQACQIC FVKSGPKLMD FTYHANIDHK CKKDILIGRI KNVEDKSWKK
IRPRPTKTNY EGPYYICKDV AAEEECRYSG HCTFAYCQEE IDVWTLERKG
AFSREAFFGG NGKINLTVFK LLQEHLGEFI FLCEKCFDHK PRMISKRNKD
NSTACSHPVT KHEFEDNKCL VHILRETTVK YSKIRSFHGQ CQLDLCRHEV
RYGCLREDEC FYAHSLVELK VWIMQNETGI SHDAIAQESK RYWQNLEANV
PGAQVLGNQI MPGFLNMKIK FVCAQCLRNG QVIEPDKNRK YCSAKARHSW
TKDRRAMRVM SIERKKWMNI RPLPTKKQMP LQFDLCNHIA SGKKCQYVGN
CSFAHSPEER EVWTYMKENG IQDMEQFYEL WLKSQKNEKS EDIASQSNKE
NGKQIHMPTD YAEVTVDFHC WMCGKNCNSE KQWQGHISSE KHKEKVFHTE
DDQYCWQHRF PTGYFSICDR YMNGTCPEGN SCKFAHGNAE LHEWEERRDA
LKMKLNKARK DHLIGPNDND FGKYSFLFKD LN
```

untreated
+ 5-fold SAMT-247
