# Supplementary material for: Global Proteomics Investigation of SAMT-247 Targets: An Antiviral Thioester that Acetylates Zinc Finger Proteins
Source: bioRxiv. 2026 Apr 30:2026.04.28.721345. Preprint. [Version 1] doi: 10.64898/2026.04.28.721345 (PMC13142373; doi:10.64898/2026.04.28.721345)
Supplement: Supplement 4 [file media-4.pdf]

Supplemental Figure 1. A) Melting curve from TPP analysis of RU1C in the absence (blue) and presence (red) of SAMT-247. Data for replicate 1 is shown as a circle and for replicate 2 as a triangle. The black line indicates the null hypothesis of no change. B) Melting curve from TPP analysis of RNPS1 in the absence (blue) and presence (red) of SAMT-247. Data for replicate 1 is shown as a circle and for replicate 2 as a triangle. The black line indicates the null hypothesis of no change. C) Residual sum of squares analysis of all significantly shifted proteins.

Supplemental Figure 1

A

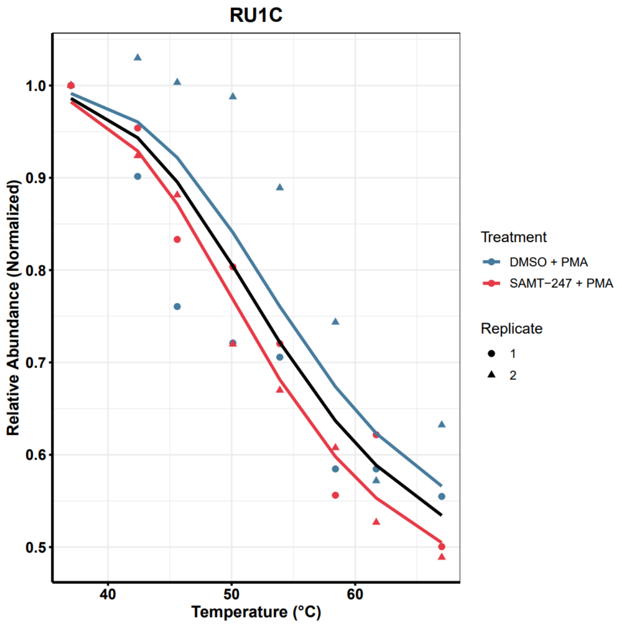

B

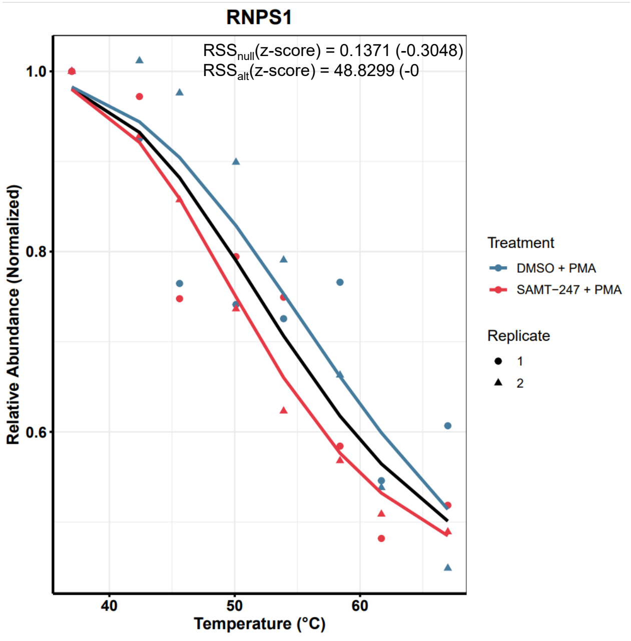

C

Reactive	Non-reactive	$RSS_{null, z\text{-}score}$	$\Delta RSS_{alt, z\text{-}score}$
DDB2	AGO1	0.073151	0.086975
POP5	AGO1	0.021759	0.040055
HNRNPH2	AGO1	0.062563	0.063618
ATP2A3	TMEM165	0.044688	0.040663
DCTN2	CAPZA2	0.003414	0.004971
MVB12A	CHMP2B	0.009969	0.010201
DPH2	GUF1	0.015809	0.028246
FLOT1	RAB35	0.028221	0.027701
PRDX3	FTL	0.064912	0.085739
GTF2E1	POLR3C	0.004177	0.009862
ZHX2	HBD	0.008072	0.045685
MARCHF6	HM13	0.006819	0.015847
RPL36AL	HMGN2	0.095891	0.082728
MARCHF6	SEC62	0.023724	0.023671
SAMM50	MRPS16	0.03459	0.005604
MVB12A	MVB12B	0.056408	0.077117
VPS28	MVB12B	0.045948	0.02743
SYAP1	PUDP	0.039878	0.044195
SNRPC	RNPS1	0.037887	0.049313
VPS16	TGFBRAP1	0.036272	0.033474
VDAC2	TSPO	0.076009	0.080874
